# Comparison of the Surgical Treatment for Strabismus According to Its Type: Esotropia Versus Exotropia

**DOI:** 10.3390/jcm15020795

**Published:** 2026-01-19

**Authors:** Antonio Martínez-Abad, Ana Siverio-Colomina, Maria Alejandra Amesty, Rosa Díez-de-la-Uz, Mario Cantó-Cerdán

**Affiliations:** 1 VISSUM, Miranza Group, C/Cabañal 1, 03016 Alicante, Spain; anaisabel.siverio@vissum.com (A.S.-C.); alejandra.amesty@vissum.com (M.A.A.); rosa.diez@vissum.com (R.D.-d.-l.-U.); mario.canto@vissum.com (M.C.-C.); 2 Grupo de Investigación en Optometría (GIOptom), University of Alicante, 03690 Alicante, Spain

**Keywords:** strabismus, ocular deviation, esotropia, exotropia, surgical treatment

## Abstract

**Background**: The direction of deviation in strabismus may influence the predictability of the surgical procedure, but this factor remains insufficiently investigated. The aim of this study was to compare postoperative changes in ocular deviation, measured by video oculography, following surgical treatment in patients with concomitant exotropia and esotropia. **Methods**: A prospective longitudinal study included 49 patients with horizontal strabismus. All patients underwent an eye examination before and after surgery, with ocular deviation measured in nine gaze positions using video oculography. Preoperative and postoperative results were analyzed separately for esotropias and exotropias to assess surgical efficacy in both conditions. **Results:** Ocular deviation significantly improved after strabismus surgery in both esotropia and exotropia across all nine gaze positions (*p* < 0.05). The greatest improvement was observed in the primary position, with an efficacy rate of 75% in exotropia (mean reduction of 14.93 prism diopters) and 78% in esotropia (mean reduction of 17.50 prism diopters). Residual postoperative deviation was similar between the two types of strabismus (*p* > 0.05). In non-primary gaze positions, surgical efficacy was lower—particularly during complex eye movements—in both groups. **Conclusions:** Strabismus surgery resulted in a significant reduction in ocular deviation across all gaze positions in patients with concomitant horizontal strabismus, as objectively assessed by video oculography. Postoperative improvements were comparable between exotropia and esotropia, with the highest surgical efficacy observed in the primary gaze position. These findings support the use of objective multigaze evaluation to more comprehensively characterize postoperative alignment and to inform future assessments of surgical outcomes.

## 1. Introduction

Strabismus is a binocular vision disorder characterized by ocular misalignment, leading to visual impairment, reduced stereopsis, and adverse psychosocial consequences [1,2]. It is a common condition, affecting approximately 3% of the general population [1]. Horizontal deviations, particularly concomitant esotropia and exotropia, are the most prevalent forms of strabismus [3,4]. These conditions often present with large-angle ocular deviations, compromising binocular visual function and exerting a substantial negative impact on mental health and quality of life [5,6].

Despite the availability of non-invasive treatment modalities for strabismus management, including optical correction with spectacles, prism therapy, and structured vision therapy, surgical intervention remains necessary in a substantial proportion of cases [7,8,9]. This is especially true for patients presenting with large-angle and/or long-standing deviations, in whom conservative approaches are often insufficient to achieve satisfactory ocular alignment for binocular visual recovery and/or esthetic improvement [10,11]. Strabismus surgery is particularly critical in pediatric patients, as early ocular alignment is closely associated with optimal visual development and improved long-term binocular outcomes. However, surgical correction in adults has also been shown to provide significant functional, psychosocial, and quality-of-life benefits, contributing to a steady increase in the global volume of strabismus surgeries over recent decades [12].

The predictability of postoperative outcomes depends on multiple factors, including the type of strabismus, the magnitude and stability of the deviation, and the complexity of the surgical procedure. Previous studies have demonstrated that incomitant deviations are associated with a poorer surgical prognosis [13], while vertical deviations tend to exhibit higher reoperation rates compared with horizontal deviations [14,15]. Nevertheless, surgical outcomes in horizontal strabismus remain incompletely characterized, underscoring the need for separate prognostic analyses of exotropia and esotropia and highlighting ongoing controversy in this field [16]. Current clinical guidelines recommend distinct management strategies for esotropia and exotropia [9,10], representing a key research gap addressed by the present investigation. To date, postoperative outcomes in horizontal strabismus have been predominantly reported in the primary position of gaze, with limited objective data describing ocular alignment behavior in non-primary gaze positions.

Furthermore, concomitant deviations are often evaluated exclusively in the primary position of gaze, without systematically assessment of ocular alignment across non-primary gaze positions. This limitation is particularly relevant when using the Alternate Prism Cover Test (APCT), which, despite being the clinical gold standard, includes an inherent examiner-dependent subjective component that may increase measurement variability and introduce potential errors in surgical planning [17]. Objective eye-tracking techniques, such as video oculography (VOG) or the Hess Lancaster Screen Test (HLST), enable examiner-independent quantification of ocular deviation and allow a more comprehensive evaluation of ocular alignment across multiple gaze positions [18,19]. Consequently, there is currently no clear consensus regarding surgical behavior and postoperative outcomes across different gaze positions, particularly when assessed using objective measurement techniques in patients with esotropia and exotropia.

The aim of this study was to compare postoperative changes in ocular deviation measured by video oculography following surgical treatment in patients with concomitant exotropia and esotropia, and to evaluate the efficacy of the surgical procedure across different gaze positions in both types of horizontal deviation.

## 2. Materials and Methods

### 2.1. Participants

This prospective observational study included 49 subjects diagnosed with concomitant horizontal strabismus who underwent surgical treatment aimed at reducing ocular deviation. Participants were recruited from the ophthalmology consultation services at Vissum Grupo Miranza (Alicante, Spain). All patients provided written informed consent prior to enrollment. The study was conducted in accordance with the Declaration of Helsinki and was approved by the Ethics Committee of the Comité Ético de Investigación Médica, Instituto de Microcirugía Ocular (protocol code: 210420-175).

Inclusion criteria comprised the presence of concomitant horizontal strabismus and clinical indication for surgical correction or reduction in ocular deviation. Exclusion criteria included the presence of ocular pathologies other than strabismus, particularly conditions affecting the pupil or anterior segment, as well as insufficient cooperation during ocular deviation measurements

### 2.2. Clinical Evaluation

All participants underwent a comprehensive optometric and ophthalmologic examination at baseline to confirm the diagnosis of horizontal strabismus and to exclude other ocular conditions. The evaluation included assessment of visual acuity, objective and subjective refractive examination, and binocular vision assessment, including stereopsis and sensory status evaluation using the TNO Stereotest and Worth four-dot test, respectively.

Ocular deviation was measured in the nine diagnostic positions of gaze using video oculography (VOG; Gazelab^®^, BCN Innova, Barcelona, Spain), software version 1.9.x (2023). This portable device incorporates two infrared cameras and a laser light projection system to assess ocular alignment across the nine gaze positions. Measurements were systematically performed at a viewing distance of 1.50 m, with lateral gaze positions assessed at 17 degrees. This device has demonstrated excellent agreement with the Alternate Prism Cover Test (APCT) for strabismus measurement, supporting its clinical validity [18]. The examination was conducted preoperatively and approximately six months postoperatively to evaluate changes in ocular deviation over time.

### 2.3. Surgical Treatment

Strabismus surgery was performed according to standard clinical practice. Muscle-weakening procedures were carried out using the recession technique, which involves posterior displacement of the muscle insertion toward its origin, whereas muscle strengthening procedures were performed using the resection technique, which consists of removing of a portion of the muscle belly and tendon near its insertion [1]. Cases requiring complex surgical approaches involving more than one extraocular muscle were excluded from the analysis.

In the sample study, bilateral surgical treatment was performed in 71% of cases and unilateral treatment in 29%. Recession procedures were performed in 84% of eyes, while resection procedures were performed in 16% of eyes.

### 2.4. Statistical Analysis

As is customary in the field of binocularity, exotropia was expressed with a negative sign and esotropia with a positive sign. However, for the analyisis of strabismus magnitude, ocular deviation was considered in absolute values. Ocular deviations were expressed in prism diopters, which can be readily converted to degrees (1 prism diopter = 0.57 degrees).

Statistical analyses were performed using SPSS version 26.0 for Windows (SPSS Inc., Chicago, IL, USA). Data was assessed using the Kolmogorov–Smirnov test and revealed non-normal distributions; therefore, non-parametric tests were applied. The Wilcoxon signed-rank test was used to compare preoperative and postoperative deviations, and the Mann–Whitney U test was used to compare outcomes between esotropia and exotropia, as well as between adults (>18 years) and children (<18 years). The chi-square test was used to analyze categorical variables.

Additionally, ocular deviation data were analyzed using a linear mixed-effects model to account for the repeated-measures structure of the dataset and the non-independence of observations within subjects. Ocular deviation (in prism diopters) was used as the dependent variable. Fixed effects included Time (preoperative vs. postoperative), Gaze position (nine diagnostic gaze positions), and Strabismus type (exotropia vs. esotropia), with Age and Sex included as covariates. Subject was included as a random intercept to model within-subject correlation. Interaction terms between Time and Gaze (Time × Gaze) and between Time, Gaze, and Strabismus Type (Time × Gaze × Type) were included to evaluate whether the magnitude of postoperative change differed across gaze positions and between deviation types. Model assumptions were assessed visually. Statistical significance was set at *p* < 0.05. Per-gaze comparisons were considered exploratory and were used descriptively to support visualization of gaze-dependent patterns.

## 3. Results

### 3.1. Participants’ Outcomes

A total of 49 patients with strabismus were included in the study, comprising 25 females (51.0%) and 24 males (49.0%). No statistically significant differences were observed between sexes (*p* > 0.05). Only horizontal deviations were analyzed. Unilateral surgical treatment was performed in 14 cases (28.6%), and bilateral surgical treatment in 35 cases (71.4%), using hang-back, recession, or resection procedures.

Table 1 summarizes the descriptive statistics of preoperative variables. No significant differences in visual acuity or refractive error were observed between eyes (*p* > 0.05), confirming sample homogeneity of the sample with respect to laterality.

### 3.2. Descriptive Comparison of Ocular Deviation Preoperatively and Postoperatively

For analytical purposes, the sample was stratified according to the type of horizontal deviation, including 29 patients with exotropia (59.2%) and 20 patients with esotropia (40.8%).

Preoperative ocular deviations across all gaze positions did not differ significantly between exotropia and esotropia groups (*p* > 0.05). Table 2 presents the ocular deviation values before and after surgical treatment in the nine diagnostic positions of gaze for both groups. A statistically significant reduction in ocular deviation was observed postoperatively across all gaze positions in both exotropia and esotropia groups (*p* < 0.05).

The comparison of functional binocular variables over time revealed a significant increase in the incidence of fusion, rising from 27% preoperatively to 73% postoperatively (*p* = 0.011). With respect to stereopsis, no statistically significant differences were detected; however, a non-significant trend toward improvement was observed (52% preoperatively vs. 68% postoperatively; *p* = 0.072). No significant differences were found between the exotropia and esotropia groups regarding the magnitude of functional changes (*p* > 0.05). Age-based stratification identified 28 adults (57%) and 21 children (43%), with no significant differences observed between age groups in terms of surgical deviation outcomes (*p* > 0.05). To illustrate the gaze-dependent postoperative patterns identified by the mixed-effects model, descriptive per-gaze summaries are presented below.

### 3.3. Efficacy of Surgery in Exotropias and Esotropias

**Figure 1:**
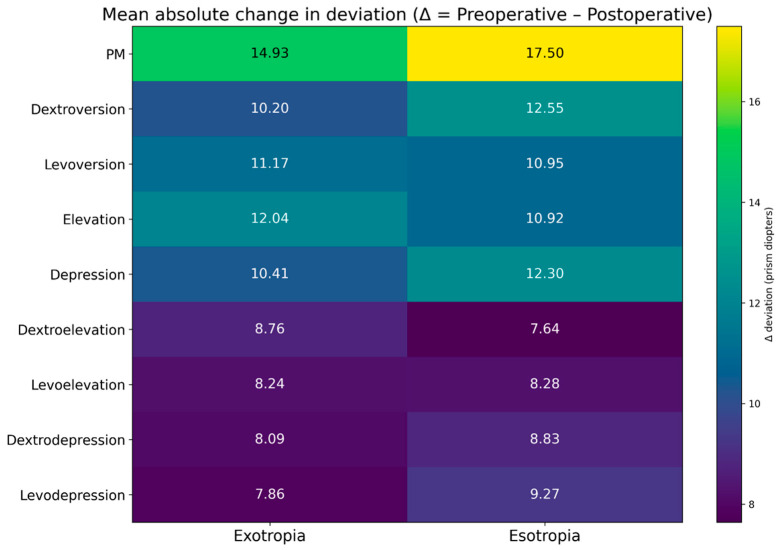
Heatmap illustrating the mean absolute change in ocular deviation (Δ = preoperative minus postoperative deviation, expressed in prism diopters) across the nine diagnostic gaze positions for exotropia and esotropia. Warmer colors indicate greater postoperative reductions in deviation. The heatmap highlights the gaze-dependent pattern of surgical correction, with the largest mean changes observed in the primary position of gaze and progressively smaller changes in non-primary, particularly combined, gaze positions. Values represent group-level mean measurements.

The absolute and relative efficacy of the surgical procedure was determined based on the magnitude and percentage change between preoperative and postoperative ocular deviation values. These results are presented in Table 3 and Table 4 and in Figure 1, which demonstrate that surgical efficacy was highest in the primary position of gaze for both exotropia (mean efficacy: 75%; Table 3) and esotropia (mean efficacy: 78%; Table 4). No statistically significant differences were observed in the mean postoperative reduction in ocular deviation across any gaze position when comparing exotropia and esotropia groups (*p* > 0.05).

Surgical efficacy was additionally assessed by reporting the proportion of patients with low residual deviation (<10 prism diopters) postoperatively. Overall, 84% of the total sample achieved this outcome in the primary position of gaze. When stratified by deviation type, success rates were 85% for esotropia and 83% for exotropia.

### 3.4. Mixed-Effects Model Analysis

To appropriately account for the repeated-measures structure of the data, a linear mixed-effects model was applied including Time, Gaze position, and Strabismus Type as fixed effects, with Subject included as a random intercept. The model revealed a significant main effect of Time, indicating a global reduction in ocular deviation following surgery across all gaze positions (*p* = 0.001). A significant Time × Gaze interaction was observed, demonstrating the magnitude of postoperative correction varied according to gaze position. Specifically, larger reductions were observed in the primary position of gaze, whereas smaller changes were detected in non-primary, particularly combined, gaze positions.

In contrast, the Time × Gaze × Strabismus type interaction was not statistically significant, indicating that gaze-dependent correction patterns were broadly comparable between exotropia and esotropia when within-subject correlations were appropriately accounted for. These findings confirm that postoperative alignment improvements in ocular alignment are gaze-dependent but similar across deviation types at the group level.

## 4. Discussion

The present study evaluated postoperative changes in ocular deviation measured objectively by VOG across the nine diagnostic gaze positions in patients with concomitant horizontal strabismus, comparing exotropia and esotropia. The main findings were: (i) strabismus surgery produced a statistically significant reduction in ocular deviation in all gaze positions in both exotropia and esotropia; (ii) surgical efficacy was highest in the primary position of gaze (75% in exotropia and 78% in esotropia), with lower efficacy in non-primary positions, particularly in combined movements such as levoelevation, dextroelevation, levodepression and dextrodepression; (iii) binocular fusion improved significantly after surgery, whereas stereopsis showed a non-significant trend toward improvement; and (iv) the magnitude of postoperative change did not differ significantly between exotropia and esotropia.

### 4.1. Postoperative Alignment Across Gaze Positions: Primary Versus Non-Primary Gaze

A consistent and clinically meaningful reduction in ocular deviation was observed in all gaze positions after surgery, with the largest relative improvement occurring in the primary position. This aligns with current clinical practice, in which surgical planning and postoperative outcome evaluation are primarily focused on alignment in primary gaze [7,9]. However, the multigaze VOG data obtained in this study indicate that residual misalignment may persist more frequently in non-primary gaze positions, particularly during combined vertical–horizontal movements.

This multigaze behavior may partially explain why some patients may remain dissatisfied despite “successful” primary position alignment and underscores the need to complement traditional primary-gaze outcome reporting with objective multigaze assessment. Previous studies focusing on objective ocular motility and eye-tracking–based assessments have emphasized the value of extending evaluation beyond primary gaze [19]. Specifically, eye trackers can capture gaze-dependent behavior more comprehensively than conventional subjective methods, providing information comparable to classical Hess–Lancaster testing while reducing examiner dependence [19]. Iwata et al. [20] reported strong agreement between objective eye-tracking–based measurements and conventional Hess screen testing across all nine diagnostic gaze directions, supporting the feasibility and clinical value of objective multigaze assessment. Similarly, Goseki et al. [21] highlighted the importance of standardized nine-direction gaze documentation for ocular motility evaluation, demonstrating improved efficiency and examiner independence.

While most validation studies of VOG systems, such as that by Narváez-Palazón et al. [22], have focused primarily on measurements in primary gaze, the present study extends this evidence by objectively characterizing postoperative ocular alignment across all nine gaze positions, providing a more comprehensive understanding of surgical outcomes in horizontal strabismus.

### 4.2. Exotropia Versus Esotropia: Comparable Efficacy Patterns

No significant differences were found between exotropia and esotropia regarding the magnitude of postoperative deviation reduction across gaze positions. This finding suggests that, when analyzed at the group level and under the present inclusion criteria (concomitant horizontal deviations; exclusion of complex multi-muscle cases), the direction of deviation alone may not be a dominant determinant of short- to mid-term motor outcomes, compared with other factors such as baseline deviation magnitude, stability, surgical dose–response, and measurement approach [13,14,15].

Nonetheless, the literature remains heterogeneous on prognostic factors in horizontal strabismus, and prior work has highlighted ongoing controversy and the need for deviation-type–specific prognostic analyses [16]. Our findings add to this discussion by providing objective, gaze-dependent postoperative data supporting broadly similar efficacy profiles for concomitant esotropia and exotropia in the sampled population.

### 4.3. Functional Outcomes: Fusion Improvement and Stereopsis Trend

Beyond motor alignment, a significant postoperative increase in fusion incidence was observed, whereas stereopsis showed a trend toward improvement. This dissociation is clinically plausible: fusion recovery may occur earlier or be more sensitive to modest alignment improvements, whereas stereopsis recovery can be constrained by long-standing sensory adaptations, amblyopia, suppression, age at onset, and duration of misalignment [2]. In adults, even when sensory outcomes are limited, psychosocial and functional benefits after alignment have been consistently reported [12], supporting the clinical relevance of surgery even when stereopsis gains are modest.

### 4.4. Added Value of Objective Measurement (VOG) for Outcome Characterization

A key contribution of this study is the objective quantification of ocular deviation across nine gaze positions. Traditional APCT, while considered a clinical reference standard, is examiner-dependent and may exhibit variability that can influence both baseline characterization and perceived postoperative change [17]. Objective technologies can reduce this variability and enable standardized multigaze comparisons.

Previous study has similarly explored technology-enabled, examiner-independent quantification of ocular deviation. For example, virtual reality-based systems have been compared against APCT, reflecting the growing interest in objective measurement approaches in strabismus care [23]. Although the device and methodology differ from VOG, such work supports the broader rationale for objective measurements. Cantó-Cerdán et al. [18] demonstrated excellent agreement and strong correlation between VOG and the APCT in subjects with esotropia and exotropia, with mean differences below clinically relevant thresholds and correlation coefficients exceeding 0.97. While that study focused on distance measurements in primary gaze, it confirmed the accuracy, reliability, and examiner-independent nature of VOG for quantifying horizontal strabismus, particularly in exotropia. The present study extends these findings by applying VOG to postoperative assessment across multiple gaze positions, providing novel information on gaze-dependent surgical outcomes that cannot be captured by conventional prism-based evaluation alone.

### 4.5. Clinical Implications

From a clinical perspective, the present findings suggest that postoperative alignment outcomes based solely on the primary gaze position may overestimate alignment quality in non-primary gaze positions in some patients. Objective multigaze assessments can offer additional descriptive information regarding gaze-dependent residual deviations, which may be useful in selected cases, such as patients reporting postoperative symptoms despite satisfactory primary gaze alignment.

Given the exploratory nature of the study, the limited sample size, and the exclusion of complex multi-muscle cases, these observations should be interpreted with caution and should not be viewed as a recommendation for routine multigaze assessment in all patients. Rather, the results highlight the potential value of objective multigaze evaluation as a complementary tool for postoperative characterization and hypothesis generation in horizontal strabismus, pending confirmation in larger and longitudinal studies.

### 4.6. Limitations and Future Directions

This study has several limitations. First, the sample size limited stratification by surgical procedure details (e.g., specific muscle(s) operated, dose, unilateral vs. bilateral strategy) and by deviation magnitude, which could reveal differential dose–response behavior. Second, follow-up was performed at approximately six months, limiting assessment of long-term stability; longer-term studies are needed to evaluate stability and reoperation risk. Kopmann et al. [24] have reported that alignment success may change over time and that reoperation rates can be substantial in some cohorts. Third, complex multi-muscle surgeries were excluded, which improves internal consistency but may limit generalizability to more complex clinical cases, representing a potential selection bias. Finally, functional outcomes were limited to fusion and stereopsis; future studies could incorporate validated quality-of-life instruments and symptom metrics to better capture patient-perceived benefit.

Overall, our findings support the utility of objective VOG-based multigaze measurements to characterize postoperative outcomes in horizontal strabismus, demonstrating significant improvement across gaze positions and comparable efficacy in exotropia and esotropia, with maximal efficacy in primary position and reduced efficacy in non-primary gaze.

## 5. Conclusions

This study demonstrates that objective video oculography can detect significant postoperative reductions in ocular deviation across all nine diagnostic gaze positions in patients with concomitant horizontal strabismus, with maximal efficacy observed in the primary position and lower efficacy in non-primary gaze positions. Comparable gaze-dependent efficacy patterns were observed in both exotropia and esotropia.

These findings suggest that postoperative alignment assessment limited to primary gaze may not fully capture residual gaze-dependent misalignment. Objective multigaze eye-tracking measurements may therefore serve as a valuable complementary tool for characterizing postoperative outcomes in research and selected clinical settings. Further studies with larger cohorts, longer follow-up, and integration of patient-reported outcomes are required to clarify the role of multigaze assessment in routine strabismus care.

## Figures and Tables

**Table 1:** Preoperative values of a total sample.

	Mean	SD	Minimum	Maximum
Age	27.49	22.34	4	87
UDVA—RE (decimal)	0.70	0.35	0.05	1.20
UDVA—LE (decimal)	0.73	0.35	0.05	1.20
Sphere—RE (D)	1.12	3.46	−7.50	10.00
Sphere—LE (D)	1.23	3.48	−5.00	10.00
Cylinder—RE (D)	−1.03	1.06	0.00	−4.50
Cylinder—LE (D)	−1.10	1.00	0.00	−3.50
CDVA—RE (decimal)	0.90	0.24	0.05	1.20
CDVA—LE (decimal)	0.91	0.21	0.17	1.20

**Table 2:** Ocular deviations before (preoperative) and after (postoperative) strabismus surgery in exotropia and esotropia groups in the nine positions of gaze noted in absolute values.

Mean ± SD	Preoperative Exotropia	Postoperative Exotropia	*p*-Value Exotropia	Preoperative Esotropia	Postoperative Esotropia	*p*-Value Esotropia
PM	19.90 ± 12.23	4.97 ± 6.20	<0.001	22.30 ± 7.76	4.80 ± 4.61	<0.001
Dextroversion	14.03 ± 12.30	3.83 ± 6.50	<0.001	17.95 ± 11.73	5.40 ± 5.53	<0.001
Levoversion	17.93 ± 13.69	6.76 ± 6.68	<0.001	17.90 ± 10.56	6.95 ± 6.38	<0.001
Elevation	18.07 ± 12.65	6.03 ± 9.41	<0.001	18.37 ± 10.21	7.45 ± 6.36	<0.001
Depression	17.93 ± 11.76	7.52 ± 8.04	<0.001	19.58 ± 11.15	7.28 ± 7.63	<0.001
Dextroelevation	17.38 ± 12.38	8.62 ± 10.14	<0.001	14.82 ± 10.12	7.18 ± 6.08	<0.001
Levoelevation	17.24 ± 14.68	9.00 ± 10.72	<0.001	15.55 ± 10.62	7.27 ± 6.34	<0.001
Dextrodepression	13.57 ± 11.12	5.48 ± 6.15	<0.001	18.73 ± 12.25	9.90 ± 9.00	<0.001
Levodepression	14.57 ± 11.79	6.71 ± 7.20	<0.001	19.27 ± 13.69	10.00 ± 8.62	<0.001

**Table 3:** Mean and percentage change in ocular deviation between preoperative and postoperative measurements in the exotropia group across the nine diagnostic gaze positions.

∆ Deviation Exotropia Mean ± SD (%)		
**Levoelevation**	**Elevation**	**Dextroelevation**
8.24 ± 14.34 (48%)	12.03 ± 11.56 (67%)	8.76 ± 9.21 (50%)
**Levoversion**	**PM**	**Dextroversion**
11.17 ± 12.33 (62%)	14.93 ± 11.16 (75%)	7.28 ± 10.26 (73%)
**Levodepression**	**Depression**	**Dextrodepression**
7.86 ± 12.60 (54%)	10.41 ± 11.12 (58%)	8.10 ± 7.48 (60%)

**Table 4:** Mean and percentage change in ocular deviation between preoperative and postoperative measurements in the esotropia group across the nine diagnostic gaze positions.

∆ Deviation Esotropia Mean ± SD (%)		
**Levoelevation**	**Elevation**	**Dextroelevation**
8.27 ± 10.55 (53%)	12.06 ± 11.74 (59%)	7.64 ± 12.32 (52%)
**Levoversion**	**PM**	**Dextroversion**
11.63 ± 11.22 (61%)	17.50 ± 10.45 (78%)	12.37 ± 13.59 (70%)
**Levodepression**	**Depression**	**Dextrodepression**
9.80 ± 14.93 (48%)	12.06 ± 11.74 (63%)	9.90 ± 14.34 (47%)

## Data Availability

All data generated or analyzed during this study are included in this article. Further inquiries can be directed to the corresponding author.

## Abbreviations

The following abbreviations are used in this manuscript:

APCT	Alternate Prism Cover Test
VOG	Video Oculography
RE	Right eye
LE	Left eye
SD	Standard deviation
UDVA	Uncorrected distance visual acuity
CDVA	Corrected distance visual acuity
PM	Primary position
